# An Alveolar Dilator

**Published:** 1883-06

**Authors:** 


### AN ALVEOLAR DILATOR.

A new instrument is described by Dr. Hagelberg, of Berlin, in the February number of the *Deutsche Monatsschrift fur Fahnheilkunde.*

The instrument is designed to dilate the alveolus in the extraction of such roots as are difficult and exceedingly painful to remove by the ordinary means. The pain produced in the use of excising forceps is very severe, and few people will submit to he operation without the use of an anæsthetic. This it is dangerous to administer to patients afflicted with heart disease, plethoric diathesis, etc. Furthermore, it is often impossible with excising forceps to remove a badly decayed or broken root, when the alveolar border is very short and the palatine arch shallow. Dr. Hagelberg narrates the incidents of a case which gave him the first idea of the instrument. He says " An hysterical lady wished to have the left superior second bicuspid and molar, and the roots of the right superior second molar extracted, with chloroform. The patient was very restless under the influence of the anæsthetic, at first stamping with her feet, and afterward, as narcosis progressed, tossing the head from side to side, and this lasted during the whole period. It was necessary for an assistant to hold her head firmly, as without this no forceps could be applied. The two teeth were removed quite easily, but the roots, especially the lingual, which was very badly decayed, could not be extracted. These gave rise to a very offensive odor and consequently demanded removal. After the patient had returned to consciousness, I very gradually pressed a thin spatula-like instrument between the alveolus and root, which dilated the walls of the socket sufficiently to enable me to get a firm hold upon the root with ordinary root forceps and so to extract it.

As a spatula can only effect dilatation of the alveolus on one side, a pair of forceps was constructed identical in their action with a pair of clamp forceps, that is, opening their beaks when the handles were pressed together. The blades of this instrument are thin enough to enable them to pass between the root and bone, when by gradually pressing the handles the alveolus is dilated.

The instrument can be obtained from W. Blume, Wilhelm Street, Berlin."

